# Does international trade and investment liberalization facilitate corporate power in nutrition and alcohol policymaking? Applying an integrated political economy and power analysis approach to a case study of South Africa

**DOI:** 10.1186/s12992-022-00814-8

**Published:** 2022-03-12

**Authors:** Penelope Milsom, Richard Smith, Simon Moeketsi Modisenyane, Helen Walls

**Affiliations:** 1 Department of Global Health and Development, Faculty of Public Health and Policy, London School of Hygiene and Tropical Medicine, 15-17 Tavistock Place, Kings Cross, WC1H 9SH, London, UK; 2 College of Medicine and Health, University of Exeter, Exeter, UK

**Keywords:** Trade and investment, Power analysis, Political economy of health, NCD policy

## Abstract

**Background:**

While there is a growing body of legally-focused analyses exploring the potential restrictions on public health policy space due to international trade rules, few studies have adopted a more politically-informed approach. This paper applies an integrated political economy and power analysis approach to understand how power relations and dynamics emerging as a result of the international trade and investment regime influence nutrition and alcohol regulatory development in a case study of South Africa.

**Methods:**

We interviewed 36 key stakeholders involved in nutrition, alcohol and/or trade/investment policymaking in South Africa. Interview transcripts and notes were imported into NVivo and analyzed using thematic analysis. We used a conceptual framework for analyzing power in health policymaking to guide the analysis.

**Results:**

Under the neoliberal paradigm that promotes trade liberalization and market extension, corporate power in nutrition and alcohol policymaking has been entrenched in South Africa via various mechanisms. These include via close relationships between economic policymakers and industry; institutional structures that codify industry involvement in all policy development but restrict health input in economic and trade policy decisions; limited stakeholder knowledge of the broader linkages between trade/investment and food/alcohol environments; high evidentiary requirements to prove public health policy effectiveness; both deliberate use of neoliberal frames/narratives as well as processes of socialization and internalization of neoliberal ideas/values shaping perceptions and policy preferences and ultimately generating policy norms prioritizing economic/trade over health objectives.

**Conclusions:**

Exposing power in policymaking can expand our own ideational boundaries of what is required to promote transformative policy change. This work points to a number of potential strategies for challenging corporate power in nutrition and alcohol policymaking in the context of international trade and investment liberalization in South Africa.

## Background

The sale of ultra-processed foods (UPFs) and alcohol is on the rise in many low- and middle-income countries (LMICs) [1, 2]. These trends are associated with an increased focus by transnational UPF and alcohol corporations on growth of markets in these countries [2–7]. Part of the expansion strategy of these industries has included strong support for trade and investment liberalization in LMICs [8, 9] as this can reduce cost of production, improve efficiency and grow sales in these new and emerging markets. As such, international trade and investment liberalization has been linked to increased consumption of UPFs (including sugar sweetened beverages) [10–22] and alcohol [2 22], with consumption increasing at faster rates in many LMICs than occurred previously in HICs [22]. Given that exposure to and consumption of these products have significant cumulative health and social impacts throughout the life-course [23, 24]^,^ expansion of transnational UPF and alcohol corporations into LMICs facilitated by trade and investment liberalization has created a major new global public health challenge.

As their attention turns to new and emerging markets, transnational UPF and alcohol corporations are increasingly motivated to ensure favourable regulatory environments (usually with minimal regulation) in LMICs. Emerging research documents the various strategies adopted by these corporations to influence public health policy [2–28]. However, more structural drivers of nutrition and alcohol non-decisions in LMICs, including the international trade and investment system, remain under-explored empirically. In recent years, trade and health researchers have considered how international trade rules may promote health policy non-decisions. This includes through legal analyses of the potential for substantive and procedural aspects of trade and investment agreements to restrict policy space to mitigate the health impacts of unhealthy diets and harmful alcohol consumption [8, 29, 30]. However, only a few more recent empirical studies have adopted a political economy perspective focusing on key actors, their interests and institutional factors [31] to understand stakeholders’ strategic responses to trade and investment liberalization and how this may affect public health decision-making [30, 32–35]. Power theory is of different disciplinary roots, but overlaps and is complementary to the political economy approach that generally seeks to understand visible (and sometimes more hidden) power in policymaking [31]. Integrating both agency and structure, power theory offers a conceptually richer basis for analysing power in health policymaking than a purely political economy approach. Power theory moves beyond visible decision-making power, and draws greater attention to hidden power (how political and economic structures can be used to control the policy agenda), but particularly invisible power- how the socialization and internalization of ideas, shape actors’ interpretation of issues and the perceived appropriate solutions [31, 36, 37]. Despite power analysis being increasingly recognised as essential to understanding public health policy processes [38–42], no trade and health policy studies, to our knowledge, have explicitly analyzed how hidden and invisible forms of power generated or facilitated by the international trade and investment system may drive public health policy non-decision-making.

We argue that exposing all forms of power- visible, hidden and invisible- active at the nexus of trade and health policymaking is essential for addressing barriers to more progressive nutrition and alcohol regulation and achieving greater trade and health policy coherence. This paper therefore applies an integrated political economy and power analysis approach to understand how power relations and dynamics emerging in the context of the modern international trade and investment regime, influences nutrition and alcohol regulatory development in a case study of South Africa. To do this, we draw on a framework we developed in earlier work for analyzing power in public health policy processes (described below).

Ethical approval for this work was obtained from the London School of Hygiene and Tropical Medicine (28 August 2018) and the University of Cape Town (12 December 2018).

### Conceptual framework

**Fig. 1:**
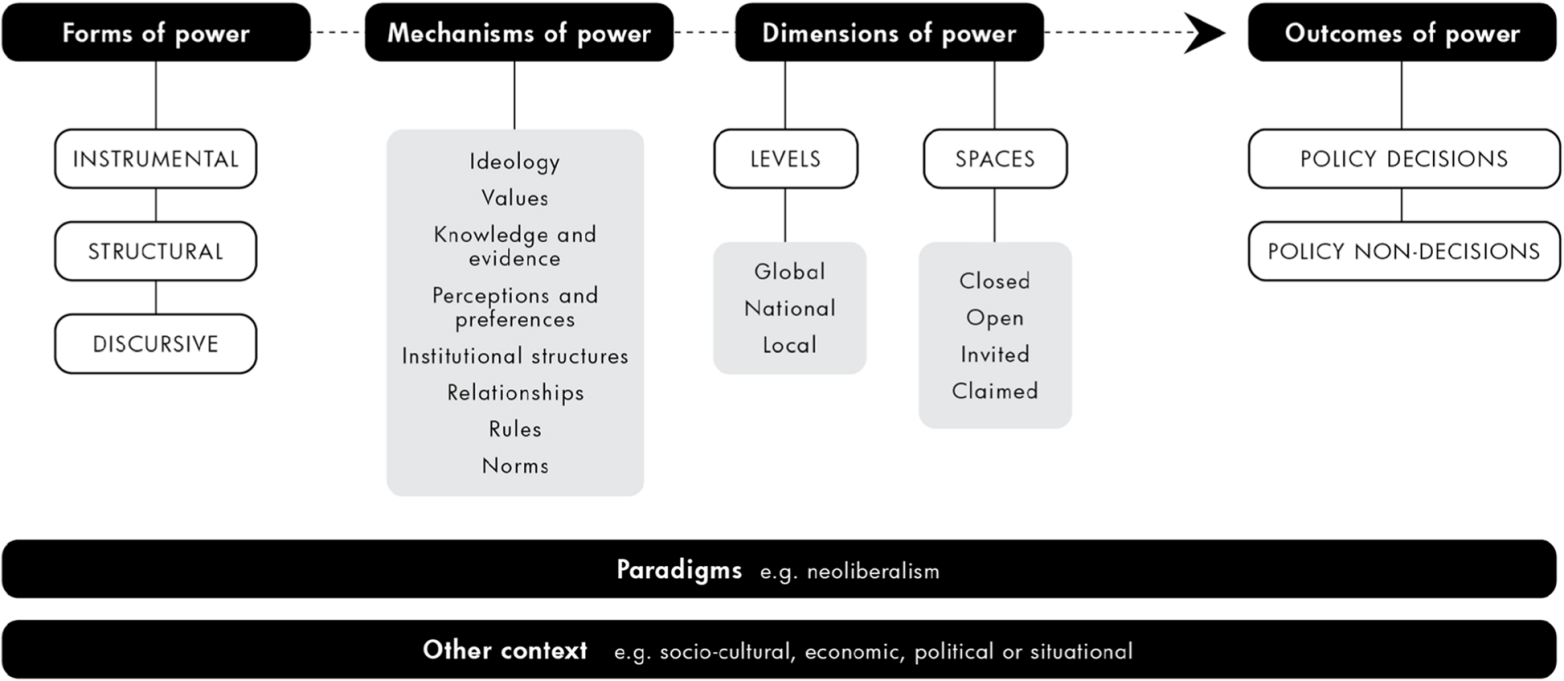
Conceptual framework for analyzing power in public health policymaking

The conceptual framework for analyzing power in public health policymaking [43] (Fig. 1) can be used as a heuristic for understanding how different forms of power operate via various mechanisms, in different spaces and across levels to influence policy decisions.

The Framework includes three *forms* of power. Instrumental power (usually most visible) focuses on the direct influence different actors have over the voluntary decisions made by formal political decision-makers. Structural power (usually more hidden) includes agenda-setting power [36]- the ability to limit who is included in decision-making spaces, whose interests are valued and the scope of alternatives for consideration [44]. Discursive power (invisible) involves holding significant problems and potential solutions outside the minds of stakeholders, including of those who stand benefit from them [45]. Discursive power usually results from the interplay between deliberate action and structural processes of socialization and internalization of accepted paradigms in societal and political values and policymaking norms.

Each *form* of power can be expressed via a number of different *mechanism* types and examples of these are provided in Milsom et al (2020), [45]. *Dimensions* of power include the different *levels* – international, national or sub-national – where power resides or is contested. Dimensions of power also include different *spaces*, defined as formal or informal opportunities where actors can ‘potentially affect policies, discourses, decisions and relationships’ relevant to their interests [45]. Spaces may be closed, invited, open or claimed by less powerful actors [45].

The *outcome* of power may be a *policy decision* taken by decision-makers to act (voluntary/involuntary and optimally/sub-optimally). Alternatively, the outcome may be a *non-decision –* a voluntary decision not to act; an involuntary failure to act; or inaction due to an ideational boundaries issue. Certain *contexts* – political, economic, socio-cultural or situational – can inhibit or activate different mechanisms of power generating different outcomes. Overarching *paradigms* determine the overall structure of power in the policymaking system.

## Methods

The ‘case’ under investigation in this case-study is recent (2012-2020) nutrition and alcohol policy non-decisions in South Africa. South Africa was selected as a rich case-study context due to its economy being relatively open to trade; role as a strategic hub from which UPF and alcohol corporations can develop new markets across Africa [6]; stated government commitment to and prioritization of NCD prevention; and its status as a regional health policy leader such that corporations may have a particular interest in ensuring a favourable regulatory environment in South Africa to prevent regional/continental policy transfer.

### Data collection

We developed a semi-structured interview guide to elicit an in-depth understanding of key actors’ ideas, values, interests and positions in relation to health and trade objectives; perceptions of the international trade and investment regime influence on diet-related non-communicable diseases (NCDs) and alcohol harm reduction policy processes; and the strategic approaches adopted by stakeholders to achieve their desired trade/health objectives. The interview guide was tested with local policy experts within academia and government and adapted accordingly before finalizing.

An initial stakeholder mapping of key stakeholders identified basis on their experience relating to food and alcohol policy issues with potential relevance to international trade or their involvement in trade/investment policy development was undertaken with input from one of the study investigators (MM), a South African Department of Health (DoH) trade and health expert. Key stakeholder identified in the mapping process were then invited to participate in an interview. Thereafter, participants were identified through snow-ball sampling.

In total 77 stakeholders were invited to participate in a one-hour semi-structured interview via email with an attached standard research information sheet. At least two additional follow-up attempts were made to contact non-responders by emails and/or phone. In total 36 gave written consent to participate in an interview and for data provided in their interview to be included in research publications, 25 did not respond and 13 declined to be interviewed, often referring us to others. An additional three stakeholders (two food corporation representatives and one from the DoH agreed to be interviewed but did not give written consent for their interviews to be included in the analysis. On review, exclusion of these interviews did not substantively alter the results reported. Table 1. presents a summary of the participants by stakeholder group.

**Table 1:** Summary of study participants

Stakeholder group	Key stakeholders invited to interview	Key stakeholder’s interviewed			
		Nutrition	Alcohol	Cross- cutting	Total included in analysis
Department of Health	17	7	1	3	10
Department. of Trade and Industry	14	0	2	6	8
National treasury	4	1	0	1	2
Department of Agriculture, Forestry and Fisheries	6	2	0	0	2
Department. of Social Development	1	0	1	0	1
Intergovernmental organisations, non- government organisations and civil society organisations	8	4	2	0	6
Multinational food or alcohol corporations (originating both from within and outside South Africa)	10	2	2	0	2 (alcohol)
Academics	11	4	2	0	5
Health Attachés for South African Embassy in Geneva or Washington DC (current or past)	6	0	0	0	0
**Total**	**77**	**19**	**10**	**10**	**36**

Thirty-five interviews were conducted with the 36 participants between April 2019 and February 2020 either in-person in Cape Town/Pretoria or via phone/teleconference. All government participants were chief or deputy directors within their respective departments with one deputy director general. Industry representatives were governance and regulatory experts, intergovernmental organisation (IGO), non-government organisation (NGO) and civil society organisation (CSO) representatives had each been engaged in recent relevant nutrition or alcohol policy processes in South Africa.

All except two interviews were recorded where detailed notes were taken instead. All recorded interviews were later transcribed in full. Following each interview, the audio recordings and/or notes were reviewed to inform adaptations to questioning for subsequent participants and for assessing the need for further interviews.

Key policy documents were also reviewed including the National Development Plan 2030, Industrial Policy Action Plan, South African Trade Policy and Strategy Framework, Strategy for the Prevention and Control of Obesity in South Africa 2015-2020, Strategic Plan for the Prevention and Control of Non-Communicable Diseases 2013-17 and The National Policy on Food and Nutrition Security for the Republic of South Africa.

### Analysis

Data were analyzed using thematic analysis. This involved the primary researcher (PM) developing codes, initially deductively, based on inter-related themes derived from the conceptual framework [43]. Additional codes were developed inductively during the analysis process. A second coder (HW) coded two of the transcripts. We subsequently reviewed each of these transcripts together to discuss and resolve any discrepancies in coding. Transcriptions were coded in NVivo (version 12.6.0). Coded extracts organized according to main themes were then transferred into Word documents to identify patterns across key informant interviews. Results are reported according to the various mechanisms of power described in the conceptual framework and identified during the analysis as relevant to this case study. Some relevant data sourced from the literature is also integrated into the results with the purpose of providing additional context to the analysis of the interview data. In practice there is considerable overlap and significant inter-dependence between many of the mechanisms presented. The analysis was reviewed by MM, a South African trade and health expert working within the DoH.

## Results

### Neoliberal paradigm and ideas

The neoliberal idea that free and open competitive markets in all areas of life will achieve economic growth and shared prosperity [46] has influenced governance and policy in most countries, although in different ways. From the 1990s in South Africa, neoliberalism took the form of trade liberalization, privatisation, state deregulation and corporate self-regulation [47]. These processes were considered necessary by economic actors to address the urgent problems of poverty and unemployment, while NCDs are perceived as longer-term challenges. As one trade policymaker commented:

“*we need to grow our economy; we need to expand our exports and that’s why we’re also entering into free trade agreements.*” [DTI1]

Trade and agricultural policy actors reflected that neoliberal policy reform has increased the influence of global markets on the agro-processing system (including food and alcohol), shaping actors’ interests and goals towards a much greater focus on expanding the production of value-added products (including processed foods and alcoholic beverages) since these provide the greatest financial return on the global market.

For alcohol and food corporations neoliberal reform including trade and investment liberalization and the country’s regional and global connectedness, have promoted their investment into South Africa as a production hub for accessing new markets across Africa [6]. Due to increased competition within South Africa and the opportunity for expansion across borders there has also been multi-nationalization of South African companies, especially regionally [48, 49]. As such, food and alcohol industries’ particular interest in exercising power over South Africa’s regulatory environment is motivated not only by a desire to profit within South Africa and open new markets, but also to prevent policy transfer across Africa, since South Africa was considered a regional and global policy leader as one health stakeholder commented:

“*Their interest is based on the fact that if they lose the fight with South Africa, then they will lose the war with the rest of the continent*” [H1]

These shifts in actor goals and interests that have emerged under an overarching neoliberal paradigm have facilitated corporate and economic actors to exercise different forms of power (via various mechanisms) to influence South Africa’s regulatory environment. It has also generated power within nutrition and alcohol policy spaces via deterministic mechanisms of socialization and internalization. These mechanisms are explored below.

### Relationships

South Africa’s alcohol industry in particular is dominated by a relatively small number of large multinational corporations (originating both from within and outside South Africa) [50]. These corporations have established highly organized networks and umbrella organizations, e.g., the South African Liquor Brand Owners Association (SALBA). There has also been an increase in foreign investment in the alcohol sector. For example AB InBev, the world’s largest beer brewing company bought out its leading rival SABMiller, originally a South African company, in the third largest corporate merger in history [47].

One alcohol advocate also reported increased co-ownership of corporations across the UPF, alcohol and tobacco industries in recent decades, although was unable to substantiate this claim in detail. The same advocate described a trend towards strategic inter-industry co-operation generating a more powerful “*collective push-back*” against undesirable regulation of their products: “*So we’re seeing that you wouldn’t only be dealing with one industry, even though one industry takes the lead”* [AA2]. For example, it was reported that the alcohol industry supported the UPF industry by pushing “*an agenda of information and education and awareness raising rather than taxes*”[AA2] to oppose South Africa’s tax on sugar sweetened beverages before it was introduced in 2018. It was considered in general that the building of inter-industry alliances would “*make them even more powerful when they have to engage with policy makers*” [AA2].

Concurrently, to achieve priority targets of economic growth and job creation, economic policymakers are focused on supporting growth in the already dominant agro-processing sector to produce exportable value-added products. One public health advocate reflected that with economic policymakers’ own performance measured against job creation targets, they are heavily incentivized to grant productively powerful, export commodity-producing corporations – both domestically-owned and multinational – with significant levels of access to and influence within economic/trade decision-making spaces. The same public health advocate commented:

“*Our current government has a very close relationship with business basically … because we have a high unemployment problem. They think the only people that can help them solve that is business, and business comes with all of these other neoliberal policies including … international trade agreements.”* [AA2]

A number of respondents from within the Department of Trade and Industry (DTI) confirmed that they actively cultivated close relationships with industry, encouraged engagement regarding any issues of concern and would go ‘an extra mile’ to get industry’s input during regulatory development. In contrast, an alcohol CSO representative commented, “*health ministries are lower down in the hierarchy … and therefore their voice has got less sway* [in economic/trade policy].” [AA1]

Specifically in relation to trade agreement negotiating processes, non-South African multi-national companies reported to feedback on South African trade policy via their home country’s governments while a domestic alcohol industry actor reportedly considered themselves part of the South African negotiating team. However, this same domestic industry actor commented that their influence over South Africa’s trade negotiating position was dependent on their contribution to job creation in South Africa and the size of the potential market for their products in the negotiating partner’s country.

The food and alcohol industry were effective in capitalizing on their perceived legitimacy, using various strategies to foster closer relationships with government and expand their opportunities to exercise both instrumental and structural power. These strategies were reported to include sponsoring/joining institutions that influence government policy; use of a ‘revolving door’ between high-level government and industry; and engaging in various private-public-partnerships.

### Institutional structures

The South African Constitution obligates consultation with all interested stakeholders, including the private sector, during all policy development processes unless international obligations determine otherwise. Following an era in which non-whites were largely excluded from decision-making processes, this obligation was considered by most respondents as paramount to the democratic process. A trade policymaker commented:

“*All stakeholders have equal opportunities. If anyone from an NGO wants to meet with me … they just write an email, and we have to engage with them. It’s the same as an executive of a huge multinational corporation*.” [DTI2]

While engagement with industry prolonged health policy processes, some health policymakers considered such engagement important to ensure a proposed regulation was technically feasible; for securing buy-in to increase likelihood of policy adoption; to increase implementation success; and due to a belief, that, having created the problem, industry must be part of the solution. However, nutrition policymakers emphasised that the DoH’s mandate is health protection and promotion which directly conflicted with industry’s profit incentive and therefore industry instrumental power over policy processes under the DoH’s mandate was limited.

However, a number of respondents identified that in part because of value-added growth objectives there was a general orientation of domestic institutional structures and processes giving greater structural and instrumental power to the DTI and industry relative to the DoH, particularly in relation to alcohol policy. As one trade policymaker pointed out:

“*we are involved in the alcohol regulation and food stuffs regulation because we are a huge exporter and importer and must be sure that domestic regulations specifically does not create unnecessary trade barriers but just address the objectives you want to achieve.*” [DTI1]

Alcohol regulation is coordinated via an Inter-Ministerial Committee on Combating Alcohol and Substance Abuse with representation from all relevant departments including the DoH, DTI, Department of Agriculture Forestry and Fisheries (DAFF) and the Department of Social Development (DSD). This mechanism for collaboration was seen by some economic actors to have elevated public health alongside trade and economic concerns relating to alcohol regulation. However, others considered existing power relations had been reproduced in this space with economic/trade interests dominating the agenda and policy decisions.

Responsibility for both alcohol and nutrition policy was divided between a number of government departments including the DoH, DAFF the DTI and the DSD. A number of respondents perceived this division of control between departments with conflicting mandates and objectives limited both the alcohol and nutrition policy agendas to primarily demand-side solutions. The DAFF was described as prioritising economic gain and export potential, and the DTI’s mandate was described narrowly by one academic as being to “*promote trade and industry*” [RN1] and to “*do what’s good for business*” [RN1].

For alcohol regulation, only alcohol labelling was under the mandate of the DoH with all other alcohol regulation coordinated by other departments with DoH consultation as required.

Existing formal governmental structures tended to limit the DoH’s power to advance health interests on economic/trade policy agendas or in decision-making. The government cluster system was established to foster an integrated approach to governance by increasing inter-departmental co-ordination on cross-cutting issues and ensuring alignment of government priorities before they are taken to Cabinet. DoH is however not included in the Economic Sectors and Employment or the International Cooperation, Trade and Security Clusters that have input on all trade and investment policy. DoH is also not included in the National Economic Development and Labour Council (NEDLAC) which provides a particularly important formal vehicle for business to negotiate with government and labour on development, financial, trade and industrial policy. As one CSO representative commented it “*is a very formal channel of access where they* [alcohol and food corporations] *are able to leverage and negotiate*” [AA2] and all relevant policy must be ‘approved’ by NEDLAC before advancing. Although NEDLAC is officially inclusive of community organisations, in practice it was not perceived by civil society actors as an accessible platform.

Further, the DoH is not formally involved in South Africa’s trade agreement negotiations, instead any consultation with health actors is on an ad hoc basis. A number of stakeholders indicated that public health issues relating to nutrition or alcohol harm reduction are not generally recognised as either relevant and/or key priorities during such negotiations. One DAFF policymaker stated, for example:

“*from where I’m sitting with bi-lateral and multi-lateral trade agreements, nutritional security doesn’t really play a major role”* [DAF1].

Other institutional factors limiting health actors’ engagement in trade and investment issues related to the lack of capacity within the DoH to analyze the public health implications of trade and investment policy and engage effectively in related discussions. As one health stakeholder pointed out:

“*public health advocates have never been trained on diplomacy, on politics, on trade and investment. So, if they are participating in these particular forums, they do not seem to have a good conceptual understanding of trade and investment dynamics*”. [H1]

### Knowledge and Evidence

#### Knowledge

Decision-makers tended to understand issues at the intersection of trade/health narrowly in terms of the direct legal implications of adopting specific health measures. Very limited consideration was given to the broader impact of trade and investment policy on the food supply or alcohol environment.

Both trade and health policymakers identified trade policy as primarily relevant to nutrition and alcohol-related harm by way of the influence international trade rules’ have the formulation of isolated demand-side health regulations including front-of-package food labelling and alcohol health warning labelling. Trade policymakers considered that the existing safeguards within health policy-relevant WTO agreements (particularly the Technical Barriers to Trade Agreement (TBT)) provided sufficient space for regulating in the interest of public health. Their general understanding was that if a health regulation was non-discriminatory, bona fide and grounded in evidence, then South Africa’s trade obligations would not pose a legal barrier to adoption. This understanding may well have contributed to the exclusion of health actors from trade agreement negotiating processes, given that South Africa generally did not at the time of this research negotiate agreements that extended beyond WTO commitments.

Very limited consideration was given to the broader linkages between trade and health in terms of how trade and investment policy determine market access (e.g., expansion of investment or imports of alcohol/UPFs) which, in turn, shapes food/alcohol environments, ultimately affecting health outcomes. For example, when asked if the General Agreement on Tariffs and Trade was relevant to alcohol-related harm one trade policymaker commented: “*That particular agreement is not relevant. It’s dealing mostly with trade in goods. It's dealing with wine and spirits and all of that so it’s not really a domain of public health”* [DTI2].

Amongst health actors, there was the general perception that the majority of alcohol was South African produced, and therefore international trade and investment agreements were not relevant for alcohol harm reduction. Nutrition policymakers within the DoH however, considered trade and investment liberalization was likely to have increased the availability of inexpensive UPFs. One health stakeholder reflected on this as a lack of coherence between investment policy and health objectives:

“*there is a disjuncture between the economic policies and … what health wants to achieve … with the obesity that we have we should be reaching a stage when we do not allow introduction of certain companies in the country because they are adding more to the existing burden … we don’t say no to anything.”* [H2]

Overall, trade policymakers’ general understanding that health regulatory autonomy was sufficiently protected under South Africa’s current trade agreements as well as a limited recognition of the broader linkages between trade/investment policy, market access and alcohol/food environments meant health and trade policymakers had limited expectation to coordinate or cooperate over trade and investment strategy or policy. These factors seemed to function as relatively powerful mechanisms for DoH exclusion from institutional structures and spaces where trade and investment policy agenda is set and decisions are made. This reflected the fact that goals to align trade, agriculture and health policies were not explicitly included in the Strategic Plan for the Prevention and Management of NCDs (2013-2017), the Strategy on the Prevention and Control of Obesity (2015-2020) or the National Policy on Food and Nutrition Security.

#### Evidence

A high standard of evidence proving both the harm caused by UPF/alcohol and the effectiveness of policy measures to address this harm was demanded by industry and economic policymakers in South Africa, but also institutionalized through WTO rules. This functioned as a mechanism of both industry structural (agenda-setting) and instrumental (decision-making) power, although this was perceived as a positive influence of industry by one health policymaker.

There was also indication that an evidence-based health policy-making norm had been internalized by policymakers and a lack of evidence (particularly for policy effectiveness) was cited as a key driver of policy non-decisions. For example, when asked why a proposed front of package nutrition labelling regulation remained voluntary and not mandatory, a health official stated: “*Because we didn’t have evidence and it’s not in Codex yet*” [DHN3].

Evidence of the deleterious health impacts of a product alone was not always sufficient to drive policy change. espite clear evidence of the health impacts (and economic cost) of alcohol in South Africa, the DTI had not supported the DoH’s Draft Control of Marketing of Alcoholic Beverages Bill proposing to ban alcohol sponsorship and marketing and restrict advertising. There were also ongoing protracted delays in adoption of the 2017 Draft Liquor Amendment Bill proposingd, among other things, to increase the drinking age to 21 and ban alcohol trade within 100 metres of schools and churches.

The evidence of the harmful effects of UPFs high in sugar was not considered by the DTI to indicate serious enough harm to warrant obstructing free trade. One trade policymaker reflected:

*“we know there is a health risk* [of sugar] *but used moderately there is not really a high risk. So, it depends on the risk of a product, you cannot … remove it from your market for health purposes unless there is overwhelming scientific evidence of the product’s risks.”*[DTI1]

Power was also constituted via different forms of evidence with economic impact assessments often carrying the most weight. In 2015 the Socioeconomic Impact Assessments (SEIA) System was introduced to address concerns that the full costs, were not always considered during policy development [51]. For public health policies SEIA must be used to consider the policy’s effect on national priorities including economic growth, investment, employment creation and equity [51]. A number of health policymakers reported the SEIA had made it increasingly challenging to get some regulations approved, one commented for example:

“*The* [impact on] *business, that’s what then we really have to look at; before we never used to look at what will be the impact on other issues besides health, part of that is trade, or investment or economic benefits*.”[DHN2]

Others reflected however, that if done properly, cost analyses (and impact mitigation) can promote health policy approval by making explicit how the economic cost of unhealthy diets and alcohol-related harm can outweigh industry’s economic contributions.

Additionally, health advocates reflected that the same rigorous evidential standard applied to health regulation is not also applied to economic decision-making. One academic for example commented that economic decisions were often based on flawed modelling or ideologically-based assumptions about ‘what work’s’ to reduce poverty.

The DoH’s very limited research budget meant the high evidential requirements created a significant barrier to policy progress and often delayed policy adoption. It is likely South Africa would, for example, have struggled to provide the inappropriately high level of evidence demanded of Australia during its WTO dispute over tobacco plain packaging. Policymakers did report however that international standards and guidelines were useful in filling this evidentiary gap to justify policy proposals. The NCD Strategic Plan for example reflects WHO ‘best buy’ recommendations for preventing diet-related NCDs. However, one health policymaker commented that ‘local’ evidence was often also required and another reflected that the international guidelines have the potential to be heavily industry-influenced (e.g. Codex Alimentarius Nutrition labelling standards).

### Perception and preference-shaping

Health advocates identified a number of factors contributing to the broad internalization of neoliberal ideas which tended to drive the individualization and medicalization of health issues in general political discourse. These include the political influence of international financial organisations particularly during the 1990s; neo-liberalization of economic education; support for and dissemination of neoliberal ideas, values and logic by the political and business ‘elite’; and the delegitimization of alternatives. Combined, these processes were thought to not only help keep system-level solutions off the agenda (structural power), but possibly also outside the minds of decision-makers (discursive power).

Frames and narratives often resonating with neoliberal ideas and values were also used by industry in relation to specific policy proposals. For example, the infant formula industry used individual choice and freedom from government interference to oppose the ban on marketing of infant formula as one IGO representative described:

“[industry] *arguments were really about* [the regulation] *restricting women's access and … almost becoming a nanny state where women can’t make decisions for themselves.”* [ML1].

The alcohol industry advanced the narrative that alcohol-related harm is limited to a minority of the population calling for targeted harm reduction interventions and the promotion of moderate and responsible drinking without impinging on the individual rights and freedoms of all citizens.

Industry also widely use economic framing to promote their interests relating to policy decisions. For example, an alcohol industry representative explained:

“*when we engage with government, we talk about our contribution to GDP, our contribution to employment...we frame it in those terms … also in terms of the foreign exchange and improving our trade balance.”* [AI1]

Industry were also reported by health advocates  to frame themselves as experts in nutrition and alcohol harm reduction and as ‘part of the solution’, including by rebranding themselves as health and socially conscious companies; aiming to appear ‘healthy by association’ e.g., funding nutrition conferences; partnering on and funding social development projects; and promoting themselves as proactive self-regulators. Further, industry frame themselves as contributing to the economic survival of the poor, keeping public support for regulation low. As one alcohol harm reduction advocate described:

“*They* [workers] *have no financial resources to buy any other product, but … the alcohol industry … capitalize on their desperation by giving them the product upfront free and they only pay for it after they’ve sold it, or they give them the fridge for free, as long as they only sell alcohol. Coca-Cola does the same.*” [AA1]

Health policymakers reported increasingly using economic framings of nutrition and alcohol-related harm as the most effective strategy for advancing proposed regulation. Although health policymakers recognise the importance of healthy food environments to promote healthy diets, framing nutrition as a food system problem requiring a trans-sectoral policy response did not dominate. One academic commented for example:

“*the NCD stuff is all framed around individual choice …* the *NCD stuff coming out from the DoH does … overstress the lifestyle elements.*” [RN2]

Processes of socialization and internalization of the accepted neoliberal paradigm, coupled with limited knowledge of the linkages between trade and health, appear to have influenced the interpretation of health issues by economic policymakers. These actors tended to emphasize unhealthy diets and alcohol-related harm as problems of individual choice, not system outcomes. They also generally interpreted food and alcohol primarily as economic commodities. For example, the increase in importation of both cheap sugar from powerful trading partners as well as UPFs was perceived as an economic threat, not a health concern.

It was only very recently that the DoH had managed to shift the DTI’s perception towards alcohol being “*a public health problem requiring a public health response*” [H4]. However, trade policymakers still tended to understand alcohol as problem of abuse by a limited group of individuals, not a wider system problem.

It’s within this interpretive context that the National Development Plan 2030 (NDP) and Trade Policy and Strategic Framework include objectives to increase investment, productivity and employment in the agro-processing sector (of which food and beverages are the two largest sub-divisions) and to open export markets for value-added processed products (including processed food and alcoholic beverages) [30, 52, 53]. For example, a trade policymaker reported:

“*one of our programs is to add more value to sugar … all the products under the agricultural sector, commodities where you can add value to, that is very important for us and there are support programmes to attract more investment and to increase manufacturing*” [DTI1]

While reducing poverty and increasingly employment have positive ‘spill-over’ effects on nutrition [30] and alcohol harm-reduction, health actors were concerned that the strategic economic approach developed to achieve this did not consider the health implications and one trade policymaker confirmed health had not been a priority. Further, this approach is in direct tension with both the Strategic Plan for the Prevention and Management of NCDs (2013-2017) and the Strategy for the Prevention and Control of Obesity (2015-2020) that include aims of taking a multi-sectoral approach to address NCDs and obesity including by ensuring the availability and accessibility of healthy food choices [54, 55]. There is currently no strategic plan on preventing alcohol-related harm.

Nutrition is a key priority in the NDP, however the focus is on direct interventions for maternal and child undernutrition with no mention of broader food supply interventions [30, 52]. Similarly, reducing alcohol-related harm is included as a health priority but the focus is on individual-level health sector interventions e.g. alcohol abuse programs [52] and health warning labels. A number of health actors recognized that the goal of value-added economic growth incentivized government to limit public health regulations so as not to obstruct industry profit-making activities.

The National Policy on Food and Nutrition Security (2014) commits the government to ensuring “*the availability, accessibility and affordability of safe and nutritious food*” for all South Africans [56] and states “*this can be achieved through the implementation of the following five pillars*”: improved nutritional safety nets (e.g., feeding programmes); nutrition education; investment in agriculture for local economic development; improved market participation of the emerging agricultural sector; and food and nutrition security risk management [56]. The policy makes no mention of UPFs in the food system nor does it consider trade or investment policy as potential levers to reduce the availability of unhealthy foods. Despite the policy including nutrition security as a goal, a number of stakeholders reflected on a tendency for policymakers to focus primarily on food security. One food researcher commented that particularly among economic policymakers, there was “*the belief that the system works*” [RN1] as long as the food system was supplying food, regardless of its nutritional quality. A DAFF policymaker conceded nutrition was less of a focus than food security and noted that: “*we have a very open and transparent market and that is how we try to solve the food security challenge*” [DAF1].

When asked whether policymakers considered how trade policy could affect the nutritional quality of the food system, the same stakeholder commented:

“*In any related* [trade] *negotiations South Africa’s nutrition security goals are not really considered, economic concerns are the primary factor considered*”. [DAF1]

### Norms

The dominant neoliberal narrative that value-added and export-driven economic growth is critical for realizing shared prosperity in South Africa had been internalized by trade and agriculture policymakers and was expressed in policymaking norms that prioritize economic/trade over health objectives. As one academic commented:

*“Things will only happen if they don’t impact job creation and … economic growth”* [RN1]

In relation to nutrition policy, a DAFF policymaker further elaborated:

*“DAFF is not so much concerned about dietary diversity. DAFF is in the business of making money. So, they look at the commodities that gives them some return … if you invest you want to have a return on capital and that … is easier achievable if you’re exporting your products”* [DAF2]

Given the evidence of serious health impacts of alcohol, policymakers within the DTI described balancing health and economic/trade objectives in policymaking:

*“government has to strike a balance … we don’t discount or under-estimate the important role in that industry and international investors play. They’re fundamental to growing our economy, they’re fundamental to employing our people so of course we do welcome the investment and we do want to ensure that our environment is conducive to that but... we also have to balance that with public interest”* [DTI2]

However, in practice the failure to adopt key alcohol regulations previously described, indicate economic concerns remain the priority.

DoH policymakers reported that the economic costs of nutrition and alcohol regulations to business and trading partners was a necessary consideration in policy development, but they strongly affirmed industry interests were not prioritized in policy decisions under their mandate. Balancing economic and health objectives was however evident in a number of nutrition policy decisions. For example, a proposed ban on marketing of unhealthy foods to children under 12 and the use of celebrity endorsements and promotions to market unhealthy food to children up to 18 was gazetted in 2014 but not progressed. The 2015-2020 Strategy for Obesity only includes strengthening voluntary advertising pledges.

South Africa’s trade obligations also drove the norm of balancing health with economic objectives as, for example, one health stakeholder commented:

*“I think the DoH tends to stay quite strong on these things* [nutrition and alcohol regulations]*. But at the same time, doesn't want to go against agreements that have been reached by the DTI. So ...it's trying to find compromises.”* [H3]

Major departures from these described policy norms have been observed in relation to problems with immediate, direct and severe health impacts. The AIDS epidemic, for example, triggered South Africa’s shift in position on the WTO’s Agreement on Trade-Related Aspects of Intellectual Property Rights (TRIPS) leading to ongoing advocacy efforts by South Africa (and others) at the WTO to limit the constraints on access to essential medicines generated by intellectual property protection under TRIPS. It is worth noting that South Africa is neither a major exporter of originator nor generic pharmaceuticals which tend to benefit from greater or reduced intellectual property rights, respectively. Similarly, for tobacco, a shift in public acceptance had forced a political normative shift towards a very proactive approach to tobacco control despite the significant economic contribution of the tobacco industry. As one trade policymaker stated:

*“We acknowledge that they play a major role in the agricultural sector, they’re huge investors … they still employ quite a lot of people ... but the policy has always been that – if these products are no longer acceptable in the public consumption and it becomes a health issue – that you motivate these farmers to invest in other crops*.” [DTI1]

## Discussion

Applying an integrated political economy and power analysis approach, this research identifies that, via various inter-connected mechanisms, instrumental, discursive and probably structural power (although this was the most difficult to identify) are active at the intersection of trade and health policy in South Africa. These different forms of power contribute to nutrition and alcohol policy non-decisions and broad incoherence between trade/economic policy and nutrition and alcohol harm reduction objectives. Recognising these forms and mechanisms of power also provides an opportunity to identify potential countervailing strategies for health actors to challenge them [45].

A strict evidence-based approach to nutrition and alcohol policy, driven by industry pressure and WTO rules, was a powerful driver of public health policy non-decisions. One potential way forward may be to advocate for an ‘evidence-informed and practice-based’ approach to nutrition and alcohol policy decisions that promotes active policy experimentation and evaluation rather than inaction [57, 58]. Increasing public health research funding will also be important. One option could be to hypothecate part of the sugar-sweetened beverage tax for this purpose. Securing major sustained funding increases however, will likely only occur once perceptions shift. Further, the norm of placing the practical burden of proving the harmful effects of products on public health actors, instead of industry being required to prove they are not harmful, should be challenged.

Generally limited knowledge or evidence of the links between trade policy and dietary change or alcohol-related outcomes meant these health issues were not perceived as particularly relevant to economic/trade policy. Strengthening the evidence base linking unhealthy diets and harmful alcohol consumption with trade and investment liberalization and communicating it effectively will be crucial [32]. Building nutrition and alcohol control advocacy group capacity and engagement with trade policy issues will be important to raise political and public awareness [29, 32, 59]. Capacity building across government departments on trade and health issues will be critical to develop a shared understanding of the linkages between trade and investment strategies/decisions and health.

Existing institutional structures tend to expand corporate structural and instrumental power and marginalise or exclude health policymakers (and civil society) from trade/economic policy spaces. Industry access to these spaces may be limited through legally binding international health agreements. Establishing or leveraging existing mechanisms for cross-departmental collaboration and coordination will be important to ensure health actors have access to these policy spaces. However, this should also be accompanied by a ‘health in all policies’ approach that explicitly mandates all government departments to ensure systematic consideration of health (including nutrition and alcohol harm reduction objectives) when developing their goals, strategies and policies . This mandate will also need to be replicated in trade bodies at both the regional and global level [11].

The perceived contribution of private industry to economic growth gives industry significant access to and influence within trade and health decision-making spaces [33]. Reducing UPF and alcohol industry influence requires challenging the invisible power of the internalized economic policymaking norm that prioritizes value-added export-driven economic growth over health as a development imperative.s. Strategies that shift existing perceptions will be necessary to achieve this. These include, for example,making industry economic contribution via sales of harmful products both publicly and politically unacceptable as has been achieved for tobacco in many countries including in South Africa, although issue complexity makes this a formidable challenge in the areas of nutrition and alcohol harm reduction. However, transferable lessons from tobacco control include  for example, working with communities and the large periphery of small-scale retailers to understand how food and alcohol corporations’ behaviour is both economically and health harmful and, at the same time, ensuring healthy alternative employment is available.

Use of frames and narratives is another key strategy to challenge the invisible power of internalized economic policy norms. This includes more actively advancing socio-ecological or system-level (as opposed to individual) framing of product consumption and the related health impacts. Using ‘governance for health’ framing embraces policy areas/actors (e.g., trade, agriculture and social development) not explicitly healthoriented but that create the system drivers of unhealthy diets and alcohol-related harm which may help these actors ‘see themselves’ as part of the solution. Additionally, using frames that highlight the direct and severe impacts of prioritizing economic/trade objectives over health (e.g., reframing NCDs as an epidemic) and human-rights and child protection framing may be helpful. Exposing the interests and values behind industries' own framing strategies can also be useful. The Covid-19 pandemic has provided a window of opportunity to shift existing policy norms with previously inconceivable policy being adopted including an alcohol ban in South Africa during lockdown [60].

By enhancing the workings of free market capitalism, neoliberalism has helped shape the interests that ultimately underpin many of the mechanisms of power identified in this research that contribute to nutrition and alcohol policy non-decisions and weak policy coherence for health. This supports other findings that neoliberal ideas may constrain policy action for NCDs [32, 46, 61–63]. We argue therefore, that potentially the most important action for public health advocates and civil society groups must be to challenge neoliberalism and the capitalist economic system underlying it by repeatedly exposing their flaws and effectively communicating viable alternatives.

### Limitations

Minimal participation by high-level political actors may have limited our access to data on the political dimension of NCD prevention policymaking. That said, access to these policy actors does not necessarily mean they would have disclosed relevant information due to both formal and informal confidentiality rules. Food industry representatives also declined to be included in the research. The analysis may also be restricted due to nondisclosure of relevant information by interviewed stakeholders. Finally, the single case study design limits the generalizability of the research findings.

## Conclusions

This research contributes an early example of applying an integrated political economy and power heuristic to empirical health policy process analysis. A key value of this approach is that by exposing all forms of power in policymaking, the ideational boundaries of what is required to promote healthy policymaking are expanded. This work points to strategies for challenging mechanisms of power in nutrition and alcohol policymaking that together offer a starting point for developing a comprehensive strategy to promote coherent and transformative policy action on unhealthy diets and alcohol-related harm.

## Data Availability

The data that support the findings of this study are available, with some restrictions applied, on reasonable request from the corresponding author [PM]. The data are not publicly available due to them containing information that could compromise research participant privacy/consent.
